# Antibodies and Derivatives Targeting DR4 and DR5 for Cancer Therapy

**DOI:** 10.3390/antib6040016

**Published:** 2017-10-25

**Authors:** Agathe Dubuisson, Olivier Micheau

**Affiliations:** 1 University Bourgogne Franche-Comté, INSERM, LNC UMR1231, F-21079 Dijon, France; Agathe.Dubuisson@u-bourgogne.fr; 2 CovalAb, Research Department, 11 Avenue Albert Einstein, 69100 Villeurbanne, Lyon, France; 3 INSERM, UMR1231, Laboratoire d’Excellence LipSTIC, F-21079 Dijon, France

**Keywords:** TRAIL, death-receptor targeting, ligand, cancer therapy, apoptosis, antibody, bi-specific, antibody drug conjugate, chimeric antigen receptor, scFv

## Abstract

Developing therapeutics that induce apoptosis in cancer cells has become an increasingly attractive approach for the past 30 years. The discovery of tumor necrosis factor (TNF) superfamily members and more specifically TNF-related apoptosis-inducing ligand (TRAIL), the only cytokine of the family capable of eradicating selectively cancer cells, led to the development of numerous TRAIL derivatives targeting death receptor 4 (DR4) and death receptor 5 (DR5) for cancer therapy. With a few exceptions, preliminary attempts to use recombinant TRAIL, agonistic antibodies, or derivatives to target TRAIL agonist receptors in the clinic have been fairly disappointing. Nonetheless, a tremendous effort, worldwide, is being put into the development of novel strategic options to target TRAIL receptors. Antibodies and derivatives allow for the design of novel and efficient agonists. We summarize and discuss here the advantages and drawbacks of the soar of TRAIL therapeutics, from the first developments to the next generation of agonistic products, with a particular insight on new concepts.

## 1. Introduction

One of the most prominent hallmarks of cancer cells is their ability to escape apoptosis, a programmed cell death process that occurs both during embryonic development and in adults, to remove unwanted cells [1]. Attempts to induce tumor cell death drove intense worldwide research, starting from conventional chemotherapy in the mid-20th century [2] to novel selective approaches, including targeting of TNF (tumor necrosis factor alpha) receptor superfamily members. As early as 1975, the discovery that TNF exhibits potent killing properties [3] unveiled the identification of endogenous cytokines displaying antitumoral properties. Unfortunately, TNF presented significant inflammatory toxicity in clinical trials [4]. In the late ‘80s, the independent discovery of two monoclonal antibodies, α-APO-1 and α-Fas [5,6], able to trigger apoptosis and induce tumor regression, fostered major interest in the identification of TNF-related family members [7,8], including receptor Fas/CD95, to which α-APO-1 and α-Fas bind specifically. Several other homologous proteins, displaying pro-apoptotic properties, have soon after been discovered, such as FasL, the Fas/CD95 cognate ligand, or TRAIL (TNF-related apoptosis inducing-ligand) and its agonist receptors [7,9,10,11,12,13]. While FasL or antibodies targeting Fas/CD95 exhibit potent pro-apoptotic capabilities, their use in the clinic was early compromised by the finding that the α-Fas monoclonal antibody induces fulminant and lethal hepatotoxicity in vivo [14]. On the contrary, however, TRAIL, early on, was found to be safe in animal models, and to display tumoricidal activity [15].

TRAIL, also known as Apo2 ligand, is a TNF family member discovered in 1995 [9]. This type 2 transmembrane protein has attracted the interest of scientists because of its capacity to specifically engage apoptosis of tumor cells, regardless of their P53 status [16]. TRAIL can induce programmed cell death in a wide range of cancer cells without harming normal tissues, thus exerting limited toxicity [17]. This specificity makes it a very promising anticancer agent. The other main advantage of TRAIL is its independence towards P53, a tumor suppressor gene, whose integrity is, most of the time, essential for conventional chemotherapeutic drugs to cure patients suffering from cancer [2]. Keeping in mind that mutations or alterations of P53 are often occurring during oncogenesis and associated with resistance to chemotherapy, TRAIL has thus been suggested to be able to exert its tumoricidal activity in a broader scope. TRAIL is present as a trimer at the cell surface of activated immune cells, where it plays an important role in tumor and viral immune surveillance [18,19,20]. Together with the soar of biopharmaceuticals and biotechnology engineering, the finding that a natural cytokine, such as TRAIL, could induce selective tumor cell death prompted great optimism to find the “magic bullet” to cure cancer. While the first generation of TRAIL agonist compounds did not present clinical efficacy [16], recent studies on TRAIL signal transduction allowed a better understanding of its machinery and requirements, leading to the development of a tremendous number of derivatives displaying increased pharmacokinetics and efficacy, including single-chain variable antibody fragment (scFvs), bispecific antibodies, chimeric antibody receptors (CARs) or conjugated derivatives, all of which are discussed in this review.

## 2. Apoptosis Induced by TRAIL

**Figure 1:**
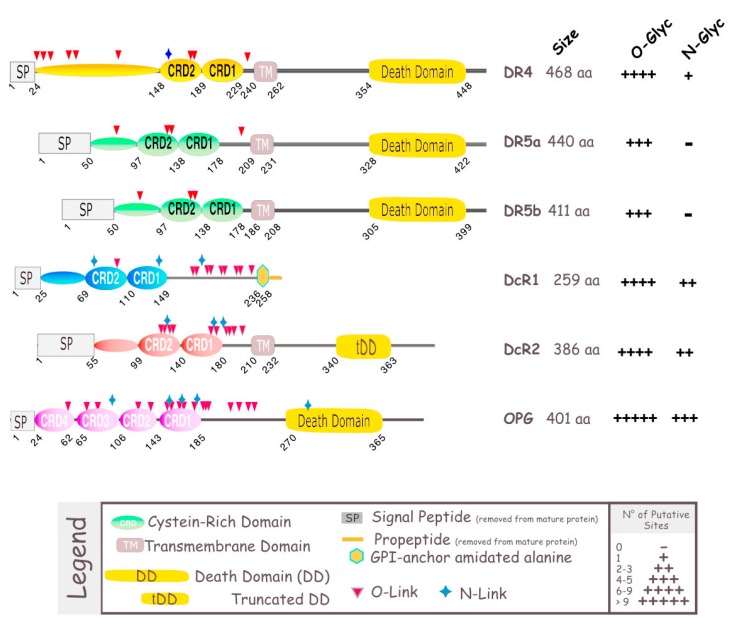
Schematic representation of TRAIL receptors. TRAIL-R1/death receptor 4 (DR4), TRAIL-R2/death receptor 5 (DR5), act as agonistic receptors, owing to their intracellular death domain (DD), through which they transmit an apoptotic signal. The other two membrane receptors, TRAIL-R3/decoy receptor 1 (DcR1) and TRAIL-R4/decoy receptor 2 (DcR2), act as antagonistic/regulatory receptors, due to their lack of functional DD. Osteoprotegerin (OPG), contains a DD, but is unable to trigger apoptosis as it lacks a transmembrane domain (TM), and is therefore a soluble receptor. DR4 and DR5 have been found to be *N*- and *O*-glycosylated, respectively [22,23,24]. DcR1, DcR2 and OPG, alike, as most members of the TNF superfamily [37], contain putative glycosylation sites, which are depicted as blue stars or red triangle. The size of these receptors is shown on the right-hand side.

**Figure 2:**
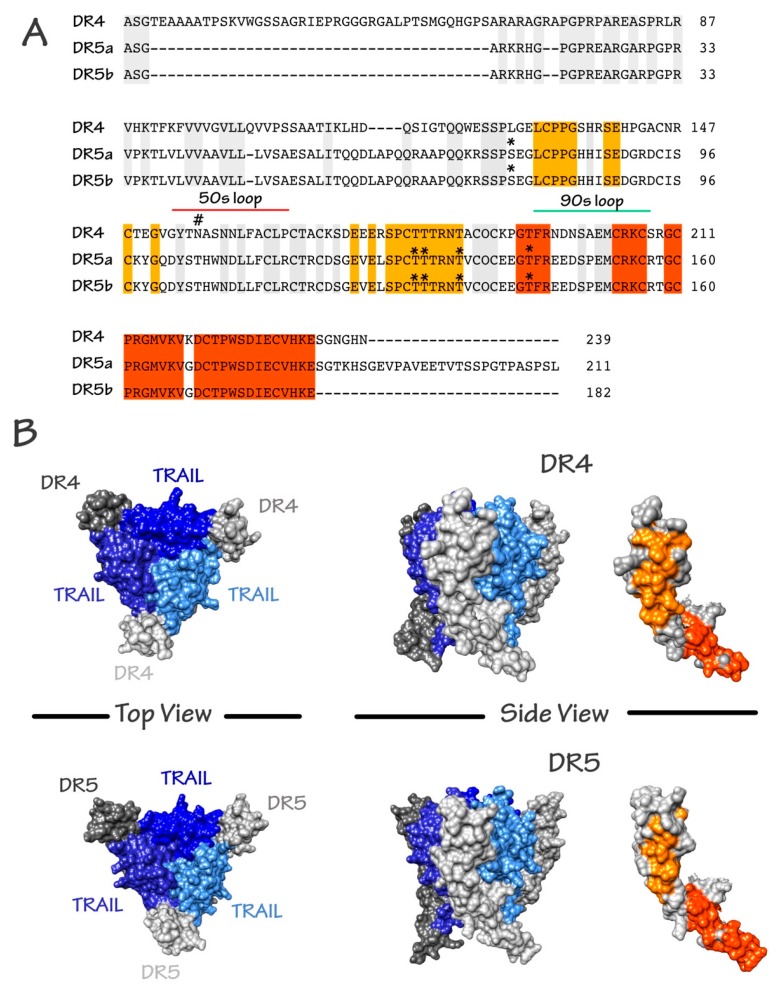
Schematic representation of the amino acid composition of TRAIL agonist receptors and crystallographic structure of the TRAIL/receptor complexes. (**A**) Alignment of DR4, DR5a, and DR5b. Sequence identity is shown. Orange and red patches correspond to shared surface residues; (**B**) Surface representation of trimeric TRAIL with DR4 and DR5 from the crystallographic structures 1D4V [42] and 5CIR [43], respectively. Top and side views are shown. Shared surface residues between DR4 and DR5 are shown by the two patches (orange and red). Buried or non-shared residues are shown in grey. In the trimeric representations, TRAIL monomers are shown as cornflower, medium, and dark blue. DR4 or DR5 monomers are shown in grey. DR4 and DR5 *N*-glycosylation and *O*-glycosylation sites are depicted with the following symbols # and *, respectively.

TRAIL induces cell death by binding to its agonist receptors. To date, five TRAIL receptors have been identified in humans: DR4, DR5, DcR1, DcR2, and osteoprotegerin (OPG) (Figure 1). These receptors, like all members of the TNF super family, are characterized by an extracellular domain, rich in cysteines [21], which are essential for the binding of their cognate ligand. Only two of them, DR4 and DR5, harbor the death domain (DD), a stretch of ~90 amino acids (aa), required and sufficient to activate the apoptotic machinery; they consequently represent promising targets for cancer treatment. DR4, is a *N*-glycosylated protein of 468 aa, also known as tumor necrosis factor receptor superfamily member 10A, CD261, Apo2, TNF-related apoptosis-inducing ligand receptor 1, and TRAILR-1 [22,23]. DR5 is *O*-glycosylated [24]. It is also known as tumor necrosis factor receptor superfamily member 10B, CD262, Killer/Ly98, TNF-related apoptosis-inducing ligand receptor 2, TRICK2A, and TRICKB. Although DR5 can be found as two isoforms, DR5a (411 aa) and DR5b (442 aa), their specific or differential functions remain, to date, unknown. DR4 and DR5 share 58% similarity in their extracellular domain and 65% similarity in their intracellular domain [10] (Figure 2A). The other receptors to which TRAIL is able to bind are DcR1 (antagonist decoy receptor for TRAIL/Apo-2L, lymphocyte inhibitor of TRAIL, TNF-related apoptosis-inducing ligand receptor 3, TRAIL receptor 3, tumor necrosis factor receptor superfamily member 10C, CD263, lymphocyte inhibitor of TRAIL (LIT), tumor necrosis factor receptor superfamily, member 10C, decoy without an intracellular domain, TRAIL-R3), DcR2 (TNF-related apoptosis-inducing ligand receptor 4, TRAIL receptor 4, TRAIL receptor with a truncated death domain (TRUNDD), tumor necrosis factor receptor superfamily member 10D, CD264, tumor necrosis factor receptor superfamily, member 10D, decoy with truncated death domain TRAIL-4), and OPG (osteoprotegerin, osteoclastogenesis inhibitory factor, TR1, tumor necrosis factor receptor superfamily, member 11B). These receptors cannot induce intracellular death signals, and are suspected to act mostly in normal tissues as decoy receptors for the retro control of TRAIL-mediated apoptosis [25]. Whereas DcR1 is a glycosylphosphatidylinositol (GPI) anchor receptor which is completely devoid of death domain, DcR2 possesses an intracellular domain but harbors a non-functional truncated death domain. OPG is the only antagonist receptor harboring a complete death domain, but it is expressed as a soluble receptor, due to the absence of transmembrane domain, and is therefore unable to transduce apoptosis. Nonetheless, like DcR1 and DcR2, albeit to a lower extent, OPG is able to compete for TRAIL binding, and impair TRAIL-induced cell death. Its binding affinity with TRAIL, though, is much weaker than DR4, DR5, DcR1, or DcR2 [26,27]. Moreover, OPG exhibits strong binding affinity with Receptor activator of NK-kappa-B ligand (RANKL), and plays a prominent role in osteoclastogenesis [28,29]. Contrary to DR4 and DR5 [30,31,32,33], DcR1, DcR2, or OPG have been found to be expressed in normal tissues, but are rarely found in tumor cells [34,35,36].

As mentioned above, TRAIL is naturally found as a trimer. It contains an unpaired cysteine residue in position 230, where other ligands of the family have a disulfide bridge that is essential for both stability of the trimer and pro-apoptotic activity [38]. Binding of TRAIL to its receptors induces their oligomerization [38]. Multimerization of agonist TRAIL receptors at the cell surface is the first critical event determining whether apoptosis will be fully triggered or not [39,40]. It is worth mentioning here that *N*- and *O*-glycosylation of DR4 and DR5, respectively, were found to be required for proper aggregation of these receptors and execution of apoptosis through recruitment of TRAIL DISC (death-inducing signaling complex) machinery [22,24]. Binding of TRAIL to DR4 and DR5, allow homotypic death domain (DD)-dependent recruitment of the adaptor protein Fas-associated death domain protein (FADD) and subsequent interaction with the pro-caspase-8, also associating within the DISC through death-effector domain homotypic interactions (see Shirley et al. for a comprehensive review [41]).

**Figure 3:**
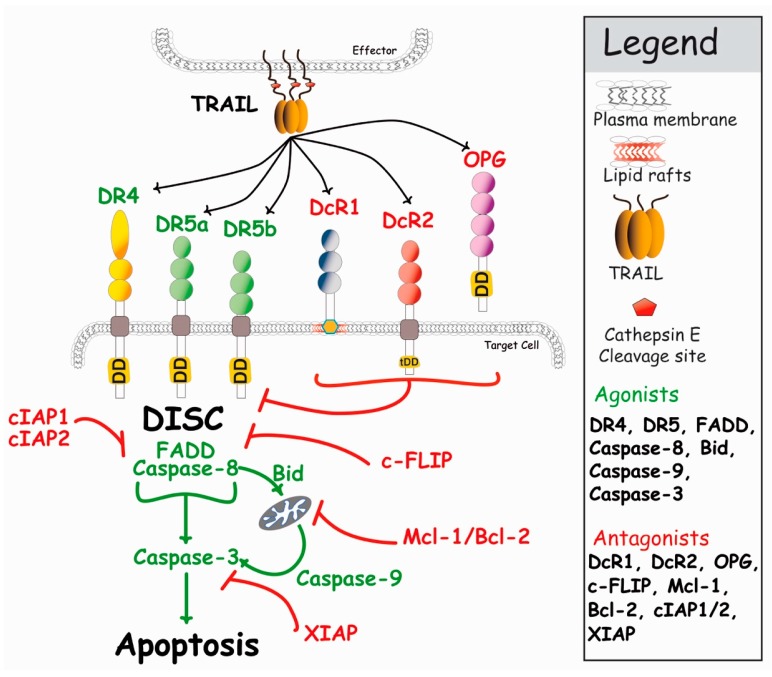
Simplified schematic representation of the main events regulating TRAIL-induced apoptosis. DR4 and DR5 agonistic receptors are able to recruit FADD and caspase-8 upon TRAIL stimulation, leading to apoptosis (see text for detail). In the presence of antagonist receptors, TRAIL-induced cell death is restrained, either due to competition for TRAIL binding (DcR1 and OPG) or steric hindrance, leading to reduced caspase-8 activation (DcR2, see Merino et al., 2006 [47], and Shirley [41]). Within the cytoplasm, the inhibitor c-FLIP, as well as Bcl-2 family members, can also restrain caspase activation and apoptosis, leading to cell resistance to TRAIL-induced cell death [48].

Formation of TRAIL DISC complex allows activation of caspase-8, and leads to the release, into the cytosol, of its functionally active subunits, enabling the cleavage of effector caspases, caspase-3 and caspase-7, whose activation by cleavage trigger execution of the apoptotic program (Figure 3). Depending on the cellular context and in particular, expression levels of inhibitors of this pathway, such as DcR1, DcR2, c-FLIP (cellular FLICE (FADD-like IL-1β-converting enzyme)-inhibitory protein), cIAP1, cIAP2, Bcl-2 (B-cell lymphoma 2) or Mcl-1 (myeloid cell leukemia 1), to cite a few, this proteolytic cascade ultimately leads to apoptosis, and to the specific killing of tumor cells bearing TRAIL agonist death receptors (DRs). When activation of the caspase-8 is not sufficient, some cells coined type II cells [44], are nevertheless able to amplify the pro-apoptotic signal through mitochondria. Also known as the intrinsic pathway, the mitochondrial pathway involves the formation of a soluble macromolecular complex known as the apoptosome [45]. This pathway, usually activated by DNA-damaging agents such as chemotherapeutic drugs, is tightly regulated by Bcl-2 proteins (see Jacquemin et al. [46]). Its activation upon TRAIL stimulation is brought by the cleavage of Bid, a BH3-only protein of the Bcl-2 family, and by caspase-8 (Figure 3), leading to cytochrome c release and to the association of pro-caspase-9 with Apaf-1 to form the apoptosome. Activation of caspase-9, in turn, results in the cleavage and activation of caspase-3 and apoptosis.

**Figure 4:**
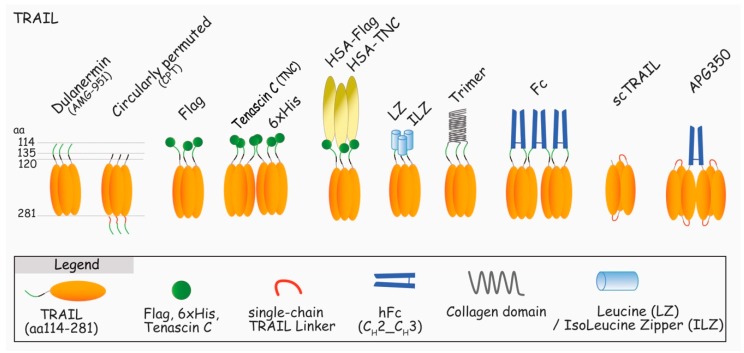
Schematic representation of the main TRAIL recombinant proteins assessed in clinical trials or used in laboratories to induce apoptosis through DR4 and/or DR5. Dulanermin (TRAIL aa114-281) [65,66], circularly permuted TRAIL (CPT, TRAIL aa135-281-linker-TRAIL aa122-135) [67], Flag-TRAIL [68], TNC-TRAIL [61], 6xHis-TRAIL [55], HSA-TRAIL [69], Leucine Zipper and Isoleucine Zipper-TRAIL [15], Trimer-TRAIL [70], Fc-TRAIL [63], Fc-sc-TRAIL [71], and AGP350 [40].

Endogenous TRAIL is naturally found associated with membranes of activated immune cells, and as such, is extremely potent in inducing apoptosis of unwanted cells [25,49,50,51,52]. Like most members of the TNF superfamily, TRAIL can be cleaved from the membranes by proteases, including cathepsin E [37], and like recombinant soluble TRAIL trimers, with the exception of a few preparations, soluble TRAIL (sTRAIL) is not able to trigger apoptosis [53,54,55,56]. To obtain biologically active TRAIL, researchers have had to use a number of strategies ranging from stabilization of the trimer with zinc [57], production of single chains [58,59], permutation of the ligand (circularly permuted TRAIL, CPT) [60], or fusion of particular amino acid or protein structures to TRAIL (i.e., 6xHis, LZ, collagen, tenascin C, or Ig-Fc) [15,61,62,63] to increase or restore TRAIL-killing capabilities (see Figure 4). Importantly, irrespective of the biological potential of TRAIL, as both DR4 and DR5 are equally able to bind TRAIL and recruit caspase-8 within the DISC, recent evidence indicates that DR4 is superior to DR5 in transducing apoptosis upon 6xHis-TRAIL binding [56], but also to “membrane-like” TRAIL, functionalized to nanoparticles [64] (also, unpublished data). Although the molecular explanation of this differential behavior remains unknown, this particularity is likely to be of interest for ongoing and future development of TRAIL derivatives aiming at treating patients in the clinic. In particular, this raises the question whether therapeutic antibody derivatives should target both receptors or not, which is the reason why we have decided to discuss both TRAIL and antibody derivatives in this review.

## 3. TRAIL in Clinical Trials

After the discovery of the attractive concept inferred from TRAIL signaling properties, attempts to translate TRAIL to the clinic was first carried out by Genentech, and next by Beijing Sunbio Biotech Co. Ltd. (Beijing, China).

### 3.1. First Attempts to Use TRAIL in Clinical Trials

Recombinant human TRAIL, rhTRAIL, has been largely tested at the preclinical level for its tumor selective killing properties and safety [17]. The good results have encouraged Genentech to develop their own construct binding to DR4 and DR5: dulanermin, rhApo2L/TRAIL, a recombinant protein encoding TRAIL from amino acids (aa) 114 to 281 (Figure 4). Unfortunately, dulanermin monotherapy did not induce sufficient overall therapeutic activity to support further clinical development [66,72,73,74,75,76,77,78]. The main limitation was proposed to be due to two main factors, its short bioavailability and inherent resistance of primary tumors to TRAIL-induced cell death [16]. Likewise, dulanermin’s half-life, ranging from 40 min to 1 h after injection, was found to be rather short, limiting, thus, its action window. The second problem is the possible resistance of the cancer cells to the treatment [79]. At the cell membrane, resistance can be due to the expression of inhibitors, including DcR1, DcR2, two antagonist receptors (DcRs) whose binding affinity with dulanermin is nearly identical to DR4 and DR5 [27]. The tumor microenvironment is a complex biological system, in which the antitumoral action of dulanermin is also likely to be compromised, irrespective of the initial sensitivity of the tumor itself. Likewise, it has been proposed recently that stromal cells harboring DcRs are also likely to impede dulanermin’s efficacy [80]. More downstream, a plethora of intracellular inhibitors can induce resistance of TRAIL after binding to its agonistic receptors [81]. The most important inhibitor being c-FLIP, whose expression in cancer cells was found to be upregulated by serum-derived active biomolecules, such as phospholipids [82]. It is therefore more than likely that its upregulation in primary tumors contributes to the lack of efficacy of dulanermin. In the same vein, pro-survival proteins including inhibitor of apoptosis proteins (IAPs), X-linked inhibitor of apoptosis protein (XIAP) or survivin, are often highly expressed in cancer cells and may counteract TRAIL-induced apoptosis as well [83]. Last, but not least, resistance may also be attributed to microRNAs, as several of them are dysregulated in tumor cells and are known to display potent pro-survival signaling potential. Likewise, miR-133a and miR-519a-3p have recently been found to induce NF-kB activation and to suppress DR5 expression [84,85].

Because conventional chemotherapeutic drugs have long been known for their ability to increase or restore TRAIL sensitivity in tumor cells [16], and even to overcome resistance induced by DcR2 [86], additional clinical trials have been set up to evaluate the efficacy of dulanermin combined with chemotherapy [16]. However, albeit well tolerated by the patients, these combinations, like dulanermin alone, did not lead to increased objective responses [16]. This lack of efficacy raised the need to develop products displaying better efficacy. It should be noted, though, that a phase III clinical trial is ongoing, aiming at evaluating the effects of dulanermin injections in the treatment of advanced non-small cell lung cancer [87].

### 3.2. Novel TRAIL Versions

In response to the disappointing results of dulanermin, several new formulations of TRAIL have been developed to improve recombinant TRAIL stability and half-life [59,88,89,90] (Figure 4). Most of the attempts have been done by fusing TRAIL to itself as single chains trimers, to single-chain variable antibody fragment (scFv), conjugating TRAIL with chemical drugs, attaching TRAIL to nanoparticles, and by expressing TRAIL on the cell surface of delivery cells. Among the numerous TRAIL fusion formulations, tenascin-C-TRAIL (TNC-TRAIL), TRAIL-Fc, and single chain-TRAIL (scTRAIL) constructs presented interesting conformational and pharmacokinetic properties. Constructs such as TNC-TRAIL were developed to stabilize the trimeric conformation of TRAIL, and were found to increase both receptor clustering and apoptosis [61]. TRAIL-Fc and scTRAIL were used to increase the pharmacokinetics properties of TRAIL, as well as its tumoricidal activity [63]. More specifically, a highly stable trimer scTRAIL (APG350) was designed as a hexavalent TRAIL. This hexameric TRAIL construct was able to induce potent programmed cell death with no need for further crosslinking of TRAIL [40], as is usually required to increase other recombinant TRAIL preparations, such as Flag-TRAIL [61,91,92].

More interestingly, Beijing Sunbio Biotech Co. Ltd. (Beijing, China) has recently developed a novel TRAIL preparation (Figure 4), described as circularly permuted TRAIL (CPT), exhibiting better stability, longer half-life, and stronger antitumor activity, as compared to dulanermin. In early phase II clinical trials, CPT was found to be relatively well tolerated and albeit limited toxicities were reported, CPT was found to elicit an antitumoral response, alone or combined with thalidomide and/or dexamethasone, in patients with relapsed or refractory multiple myeloma [60,93,94]. CPT is thus, so far, the best and most promising TRAIL derivative. However, similar to TRAIL, CPT is not only likely to suffer from resistance induced decoy receptors in patients but also likely to promote of cell migration and metastasis [95,96,97]. This implies that further assessment in preclinical animal models are likely needed, to exclude the possibility that CPT may, in a limited number of patients, promote cell-motility and/or metastasis. These limitations have, early-on, prompted the development of more specific therapies that target only DR4 and DR5.

## 4. First Antibodies in Clinical Trials

Monoclonal antibodies (moAbs) represent tools of choice for the targeting of DR4 and DR5. They clearly offer advantages for eradicating tumors. First of all, they have a longer in vivo half-life (around 14 days) than TRAIL. Second, their Fc domains allow interactions with Fc receptors (FcRs) present on the cell surface of immune cells leading to antibody-dependent cell-mediated cytotoxicity (ADCC) and complement-dependent cellular cytotoxicity (CDC). Finally, owing to their selectivity, moAbs are fully independent of decoy receptors expression, whether on the tumor cells or microenvironment. So far, seven agonistic monoclonal antibodies have been tested in clinical trials: one anti-DR4 and six anti-DR5.

**Figure 5:**
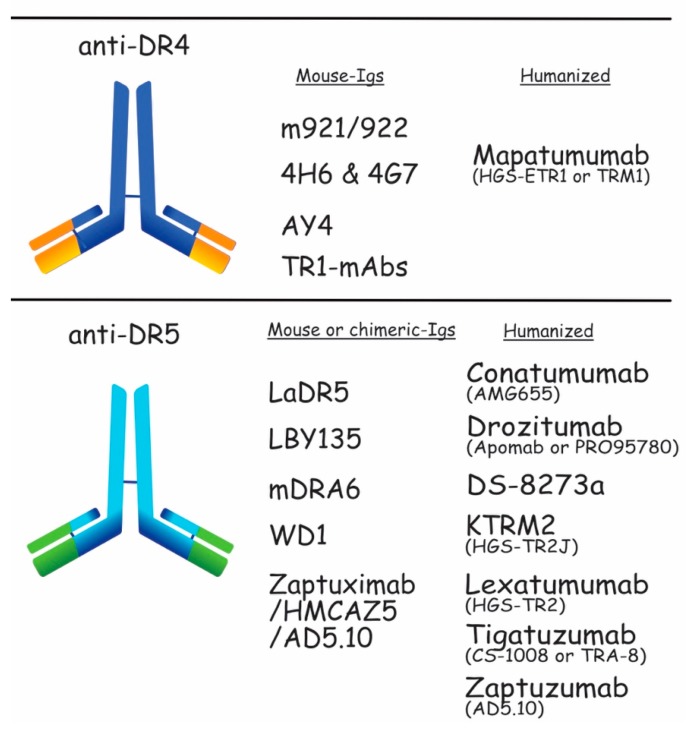
Schematic representation of the DR4 and DR5 moAb. Anti-DR4 developed in preclinical and clinical trials are shown in the upper panel. m921/922, 4H6 & 4G7, AY4, and TR1-mAbs are mouse-Igs tested in preclinical trials. Mapatumumab is a humanized-Ig assessed in clinical trials. The lower part of the figure shows the anti-DR5 developed as mouse or chimeric-Igs, which are LaDR5, LBY135, mDRA, WD1, and Zaptuximab, as well as its humanized form, Zaptuzumab. Conatumumab, Drozitumab, Tigatuzumab, KMTR2, Lexatumumab, and more recently, DS-8273a, are also humanized antibodies targeting DR5. All of them have been tested in clinical trials.

Mapatumumab, also known as HGS-ETR1 or TRM1, is the only anti-DR4 monoclonal antibody that has been evaluated in clinical trials, so far (Figure 5). Developed by the Human Genome Science (HGS) in 2005, this fully human DR4-agonistic demonstrated selective and high binding to DR4, as well as cytotoxicity efficiency [98]. Mapatumumab has been evaluated in several phase I and II clinical trials [99,100,101,102,103,104,105,106,107,108], but none of the assays met the initial objectives, prompting discontinuation of its clinical development. Other mouse moAbs targeting DR4 and displaying pro-apoptotic potential have been described in preclinical studies, including m921/922 [109], 4H6/4G7 [110], AY4 [111], and TR1-mAbs [112], but as far as we are aware of, none are being evaluated in the clinic.

As opposed to DR4, many more monoclonal antibodies against DR5 have been assessed in clinical trials. These include Conatumumab (AMG655), Drozitumab (Apomab or PRO955780), Lexatumumab (HGS-TR2), LBY135, Tigatuzumab (CS-1008 or TRA-8), and DS-8273a [113,114,115,116,117,118,119,120,121,122,123,124]. Conatumumab, Drozitumab, Lexatumumab, and DS-2873a, respectively, developed by Amgen, Genentech, HGS, and Daiichi-Sankyo, and are fully human DR5-agonistic antibodies. Conatumumab and Drozitumab exhibit effective antitumor effects against advanced solid tumors and Lexatumumab was examined in prostate cancer and bladder cancer cells [116,125,126]. DS-8273a is the latest anti-DR5 antibody assessed in the clinic. Interestingly, the first publication describing this antibody demonstrates that DS-8273a could be of use to eradicate myeloid-derived suppressor cells in patients with advanced cancer, indicating that DS-8273a could indirectly contribute to antitumor therapies [114]. Despite the fact that no objective response has been observed in this study, three additional clinical trials are ongoing, aiming at evaluating its safety in patients with advanced solid tumors and lymphomas or benefit combined to Nivolumab in patients with advanced colorectal cancer as well as unresectable stage II and IV melanoma [87].

LBY135 and Tigatuzumab are chimeric and humanized mouse–human antibodies, correspondingly developed at UAB with Daiichi-Sankyo and Novartis. LBY135 was well tolerated in solid advanced tumors, and Tigatuzumab was tested for relapsed lymphoma or solid tumors [121,127]. Alone or in combination, Conatumumab and Drozitumumab went to phase II clinical trials, whereas Lexatumumab, LBY-135, and Tigatuzumab did not get beyond phase I clinical trial [16].

Despite encouraging preclinical results, the outcomes of these clinical trials were disappointing. Most of these antibodies appeared to be relatively safe and well tolerated by the patients, but none displayed sufficient clinical benefits. At best, these moAbs induced stable disease, but none of them improved the response rates, whether used alone or combined with chemotherapy. As a consequence, and with the exception of DS-8273a, companies discontinued their development [128,129,130]. One of the reasons for the lack of clinical efficacy of these moAbs may be the inherent resistance of primary tumors to apoptosis. Apoptosis is tightly controlled by a number of activators and suppressors, whose expression levels determine sensitivity or resistance of the targeted cells, respectively [28,131,132]. After binding to its targets, moAbs initiate formation of the TRAIL DISC, but efficient signal transduction of apoptosis can be inhibited at the very early stage, at the DISC level, by c-FLIP or more downstream, at the mitochondrial level by Bcl-2 family members or cIAP1/cIAP2 and XIAP (Figure 3), leading to cell resistance [133,134,135,136,137]. Circumvention of these inhibitory checkpoints has been achieved in a number of preclinical models using conventional chemotherapy, thus restoring tumor cell sensitivity to TRAIL or agonist moAbs targeting DR4 or DR5 [16,138]. Unfortunately, neither of the combinations tested, so far, showed clinical benefits. It should be noted here that the mode of administration of the combined treatments in these early clinical trials may have also contributed to the lack of efficacy of TRAIL or anti-DR4 and -DR5 agonist moAbs. This is mainly due to the fact that these compounds are often administered into the patient either simultaneously or within short intervals, while in preclinical models, the corresponding chemotherapeutic drug is known to efficiently restore TRAIL-induced cell death, but needs to be given 24 to 72 h ahead, before TRAIL administration [16].

Another explanation for the lack of efficacy of these antibodies may be their weaker ability to trigger apoptosis. Multimerization of TRAIL agonist receptors is mandatory to transduce the apoptotic signal. As opposed to TRAIL, whose ability to trimerize TRAIL agonist receptors at the cell surface is naturally afforded by its spontaneous association as a trimer, moAbs are bivalents. This limited valency could, at least in part, explain why these monospecific antibodies are not the best formulations to target TRAIL signaling for cancer therapy. Indeed, their crosslinking is often further required to increase their antitumor potential [91,110,139,140,141,142]. Some of them, though, have been described to induce apoptosis very efficiently in the absence of crosslinking. This is the case for a DR4 bivalent antibody or KMTR2 a fully human moAb agonist targeting DR5, that induces cell death in the sensitive colorectal cell line Colo205 in a ng/mL range [143,144]. In early studies, KMTR2 has been described to be able to suppress growth of subcutaneous glioma xenografts, and prolong animal lifespan bearing intracerebral xenografts [145]. More recently, the crystallographic structure of the extracellular domain of DR5 and a Fab (fragment antigen-binding) domain derived from KMTR2, demonstrated that KMTR2 is able to induce superoligomerization of DR5 [143]. To value the use of anti-DR4 or anti-DR5 moAbs for cancer therapy, and to strengthen their pro-apoptotic potential, other rational approaches have been set up, such as increasing their valency or combining them with TRAIL [146,147,148]. But so far, none of these therapeutic options have been assessed in the clinic. However, since DR4 and DR5 are attractive targets for cancer therapy, an incredible variety of targeting molecules have been generated, which are presented below.

## 5. Novel TRAIL-Related Derivatives

Conventional antibodies are composed of two parts: two Fabs (fragment antigen-binding) linked to one Fc (crystallized fragment). Fabs contain regions highly specific to the epitope, while the Fc determines the class of immunoglobulin, and thus, the antibody potential for cell-mediated immune response. The soar of genetic engineering and our increasing understanding of TRAIL pro-apoptotic signal transduction requirements opens unprecedented opportunities to develop novel promising TRAIL-related derivatives with enhanced antitumor potential [149,150].

### 5.1. Multivalent-Based Antibodies or Peptides

**Figure 6:**
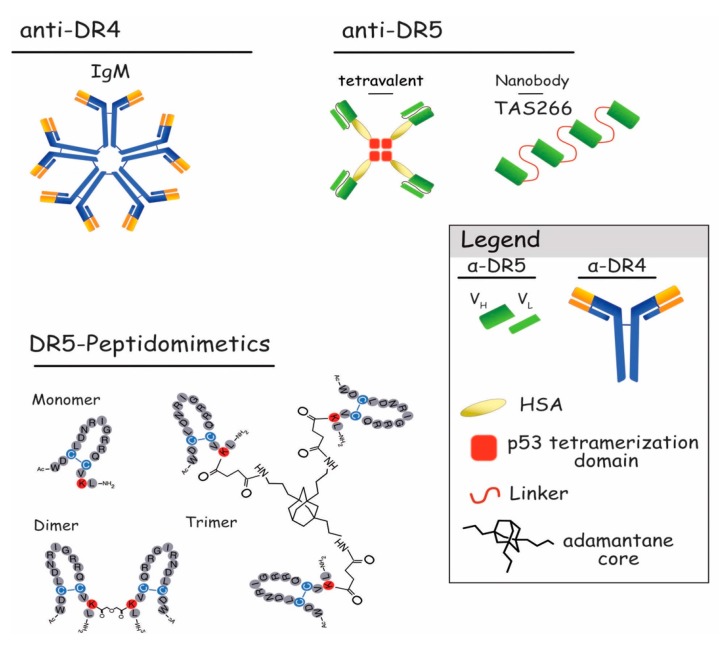
Schematic representation of DR4 or DR5 multivalent agonists. Represented on the upper left is the pentavalent IgM form of the newly described anti-DR4 antibodies [161]. The upper right shows tetravalent scFvs targeting DR5 [153] and TAS266 nanobody [154]. In the lower part, primary structures of DR5-peptidomimetics as monomer, dimer, and trimer are represented [152].

As discussed above, achieving efficient aggregation of DR4 or DR5 is probably the main bottleneck when considering TRAIL-targeting antibodies or derivatives for cancer therapy. Amongst the monoclonal anti-DR4 and DR5 antibodies described, so far, several are unable to induce tumor cell killing in the absence of crosslinking or coating to the culture plate [110,139,140,141,142]. Although antibodies assessed in clinical trial have been described to induce cell death in preclinical models, irrespective of their crosslinking, their lack of clinical efficacy is most likely due to their weaker ability to induce receptor aggregation. In line with this hypothesis, converting DR4-moAb isoforms from IgG to IgM (Figure 6) was found to enhance anti-DR4-mediated apoptosis [146], indicating that increasing moAb valency is a prerequisite to induce sufficient receptor clustering and apoptosis [151]. In the same vein, it has been demonstrated that trimeric DR5 peptidomimetics [152], a tetravalent sc-Fv:DR5 derivative antibody or a single-chain scFv:DR5 nanobody, TAS266 [153,154] display superior antitumor activities than monovalent or divalent peptides or antibodies in preclinical studies (Figure 6). Trimeric DR5 peptidomimetics, for instance, were much more efficient than their dimeric or monomeric counterpart in inducing receptor DISC formation and caspase-8 activation [152]. Whether multivalent anti-DR4 or anti-DR5 will translate to the clinic remains uncertain, so far, as it has recently been found that the scFv:DR5 nanobody, TAS266, alone, displayed hepatotoxicity in a phase I clinical assay aiming at evaluating its safety and tolerability [154]. It should be noted, however, that DR4 and DR5 exhibit distinct crosslinking requirements [91,92]. Likewise, while DR4 was found to be able to trigger apoptosis with a trimeric TRAIL ligand, apoptosis induced by DR5 required crosslinking of TRAIL trimers [92]. It should be noted here, though, that these results were obtained in the presence of cycloheximide, a protein synthesis inhibitor [155], known to impair cFLIP expression [156]. Further highlighting a differential behavior between DR4 and DR5 is the recent demonstration using isogenic cells expressing solely DR4 or DR5, that DR4 is more prone in inducing apoptosis with soluble hexameric TRAIL than DR5 [56] , in the absence of any inhibitor. DR5 remains, however, fully capable of inducing apoptosis when activated by membrane-bound TRAIL, a situation that can be recapitulated using a soluble TRAIL fused to a scFv targeting a defined antigen [92]. This strategy, which allows antigen-dependent immobilization of TRAIL at the cell surface, has largely been explored with a large panel of scFvs, and is described in the paragraph 5.2.1.

With the exception of KMTR2, Lexatumumab, and Zaptuzumab [123,124,143,144], most DR5 agonist antibodies are unable to trigger apoptosis in the absence of crosslinking. However, in vivo, they display strong antitumor activity [110], due to FcγR expressing cells that are thought to induce their crosslinking [157]. Indeed, the in vivo antitumor potential of Conatumumab, a fully human agonistic targeting DR5, was demonstrated to require FcR-mediated crosslinking to inhibit tumor growth [158,159]. FcR oligomerize antibodies that are now able to trimerize receptors, providing increasing antitumor activity. Consequently, it was thought that FcγR binding was mandatory to induce effective apoptosis. Nonetheless, additional studies shed light on the fact that apoptosis can be performed without this FcR binding. For example, scFv in the format of antibody constructs without the Fc part, thus, without the capacity to bind to FcR, showed promising results [40]. Likewise, the chimeric Fc-TRAIL-single chain hexameric variant APG 350 (Figure 4), displays potent apoptosis capabilities without interactions with FcγR-bearing myeloid cells [62,160]. Finally, the development of a new anti-DR5, Zaptuzumab, a chimeric monoclonal antibody obtained after humanization, showed promising anticancer actions without the need of crosslinking [124].

Irrespective whether co-engagement of FcR is required or not to induce suitable programed cell death, one should probably keep in mind that only co-engagement with FcγRIIB confers anti-TNFR agonistic antibodies the potential to trigger apoptosis in vivo [158].

### 5.2. Bi- and Tri-Specific Derivatives

Based on their capacity to recognize specific targets with high sensitivity, single-chain variable fragment (scFv) were developed and combined to a variety of TRAIL derivatives to induce specific killing of cancer cells. Produced with phage, yeast, and ribosome display, scFvs contain only the variable regions of the antibodies that recognize the target, without the Fc part. While maintaining specific recognition of the target, their smaller size (around 25 kDa) let them reach deeper tumor compartments, resulting in a better tumor penetration [162]. They offer the possibility to produce bispecific antibodies or chimeric proteins with the capacity to link specifically to two different targets, each of their Fab recognizing different epitopes, enabling selective antitumor therapeutic intervention [163]. These strategies allow better target specificity, as well as reduction of off-target toxicity.

#### 5.2.1. scFv-TRAIL

**Figure 7:**
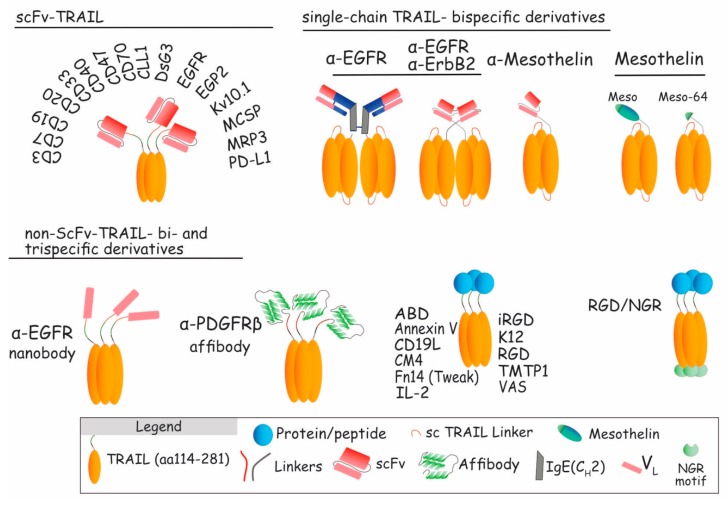
Schematic representation of scFv-TRAIL and non-scFv-TRAIL constructs. Upper left: presentation of different cancer (DsG3 [162], EGFR [149,150], EGP2 [152], Kv10.1 [153], MCSP [155], or MRP3 [156]) and immune (CD3 [163], CD7 [164], CD19 [165,166], CD20 [167], CD33 [168], CD40 [169], CD47 [170], CD70 [171], CLL1 [172], or PD-L1 [173]) antigens targeted by scFv-TRAIL constructs. Upper right: single-chain TRAIL-bispecific derivatives targeting EGFR [40,71], ErbB2 [151], and mesothelin [154]. Lower part: non-scFv-TRAIL bi- and trispecific derivatives using nanobody (ENb:TRAIL), affibody, or fusion proteins, to target cancer and immune antigens.

Various scFv-TRAIL formulations have been developed, each of which has its own advantages [88,164]. Most of the time, TRAIL-induced apoptosis is possible only in cells bearing antigens targeted by the scFv. But non-tumor cells can also be targeted to present TRAIL like a membrane-bound protein, to increase its killing efficacy. Two kinds of scFv-TRAIL were designed. The first class includes scFv-targeting antigens expressed specifically or overexpressed by cancer cells. These include scFv-TRAIL selective for epidermal growth factor (EGFR) [165,166], Erb-B2 receptor tyrosine kinase 2 (ErbB2) [59], epithelial glycoprotein-2 (EGP2) [167], Potassium channel voltage gated, subfamily H, member 1 (Kv10.1) [168], mesothelin [169], melanoma-associated chondroitin sulfate proteoglycan (MCSP) [170], or multidrug resistance protein 3 (MRP3) [171] (Figure 7). Dual targeting not only increases selectivity towards a wide range of tumors, but also affords bystander killing effects via TRAIL activity [172]. The scFv:EGFR-TRAIL is an example of such construct. EGFR is a tyrosine kinase receptor (TKR) whose expression in solid tumors is often associated with tumor development and progression [173]. scFv:EGFR-TRAIL converted soluble TRAIL into a membrane-bound form, allowing efficient apoptosis in a series of EGFR-positive tumor cell lines [166]. Based on the same principle, constructs such as ENb:TRAIL, another EGFR-targeting nanobody fused to TRAIL molecule, displayed potent antitumor activity towards cells normally resistant to EGFR inhibitors alone, or to TRAIL. In vitro and in vivo, ENb:TRAIL was able to block EGFR signaling while inducing TRAIL-mediated apoptosis of targeted cancer cells [174]. Interestingly, fusing the scFv anti-EGFR to single-chain TRAIL (scTRAIL, see Figure 7), instead of a single monomer of TRAIL, further increased the tumor killing efficiency of the scFv-TRAIL variant by 10-fold [175], highlighting the importance of TRAIL valency for its efficacy. In the same vein, an anti-EGFR diabody linked to the scTRAIL was found to exhibit strong antitumor potential both in vitro and in vivo [71,176].

ErbB2, another TKR of the same family, is also widely expressed on human tumor cells [177]. An anti-ErbB2 scFv fused to scTRAIL was found, in vitro and in vivo, to exhibit enhanced therapeutic activity as compared to scTRAIL, alone [59]. Moreover, this single chain-TRAIL variant displayed better pharmacokinetics properties compared to TRAIL, with a half-life increased by 2 to 4-fold. Other scFv-TRAILs were assessed, showing increased antitumor potential, such as EGP2, also known as epithelial cell adhesion molecule (Ep-CAM) overexpressed in carcinomas [167], the voltage-gated potassium channel Kv10.1 expressed in 70% of tumors of different origin, but not in normal cells [168], the melanoma-associated chondroitin sulfate proteoglycan (MCSP), expressed in melanoma [170], the multidrug resistance protein 3 (MRP3), expressed in glioblastoma [171], or DsG3, one of seven desmosomal cadherins that mediate cell–cell adhesion in desmosomes, whose expression has been found to be increased in primary cancer cells and associated clinical stage [178].

A number of other TRAIL bispecific constructs selective for the albumin binding domain ABD [179], annexin V [180], CM4, a small cationic linear α-helical peptide selective for tumor cells [181], Fn14 [182], integrins RGD [183,184,185] and iRGD [186], tumor molecular targeted peptide 1 (TMTP1) [187], or vasostatin (VAS) [188], have also been described, including a trispecific recombinant TRAIL protein harboring both an RGD- and an NGR-binding motif [189], all of which bind more or less specifically to tumor cells, and displaying increased TRAIL-mediated apoptosis rates. Last, further pinpointing to the growing interest for TRAIL-based therapies, an affibody targeting PDGFRβ [190], as well as scFV targeting mesothelin [169], a glycosylphosphatidylinositol anchored glycoprotein known to bind to the mucin MUC16, mesothelin itself or its MUC16-minimal-binding domain [191] fused to scTRAIL, were also described to display potent antitumor potential.

The second kind of scFvs or TRAIL-recombinant proteins target immune cell antigens, such as CD3 [192], CD7 [192,193], CD19 [194,195], CD20 [196], CD25 [197], CD33 [198], CD40 [199], CD47 [200], CD70 [201], CLL1 [202], or PD-L1 [203]. Those constructs have been generated with the aim of hastening and reinforcing immune responses by four different ways. As mentioned above for solid tumors, the first objective was to target hematological malignancies. For this, CD7, CD19, and CD33 scFv fused to TRAIL, or CD19L fused to TRAIL constructs, were produced to target and kill selectively immune cancer cells [193,194,195,198]. The second and third approaches, respectively, activate immune cells or block inhibitory signals. Those bifunctional fusion proteins confer dual pro-apoptotic signaling capacity by bringing immune cells in close proximity with tumor cells. As an example, scFv-CD40-TRAIL has the capacity to stimulate both dendritic cells (DCs) and to trigger apoptosis of DC-targeted cells [199]. Likewise, the recombinant IL2 protein fused to TRAIL targets and activated IL2 receptors overexpressed in most hematological cancers, inducing higher expression of CD25, and leading to activation of natural killer (NK) and T effector cells [197]. A particularly interesting key example of advanced TRAIL derivative is scFv-PD-L1:TRAIL [203]. This bispecific scFv arms or loads immunosuppressive PD-L1 expressing myeloid cells (as Dcs and macropages) with TRAIL molecule. Through the competitive binding to PD-L1, it inhibits, on the one hand, PD-1/PD-L1 interaction, restoring thus antitumoral immunity, and since it loads the immune cells with recombinant TRAIL, it also affords, on the other hand, TRAIL-mediated apoptosis of the targeted tumor cells. Similar to scFv-PD-L1:TRAIL, targeting CD47 or CD70 block the “don’t eat me” inhibitory signals, restoring tumoricidal activity of immune cells and inducing TRAIL-dependent pro-apoptotic signal [200,201]. Lastly, cell-based therapies targeting CD3, CCL1, or CD20 arm T-cells [192], granulocytes [202] immune cells or mesenchymal stem cells [196], respectively, with high levels of cell surface TRAIL, have also been found to efficiently induce apoptosis of immune-resistant cancer cells.

#### 5.2.2. Unconventional and Bispecific Antibodies

**Figure 8:**
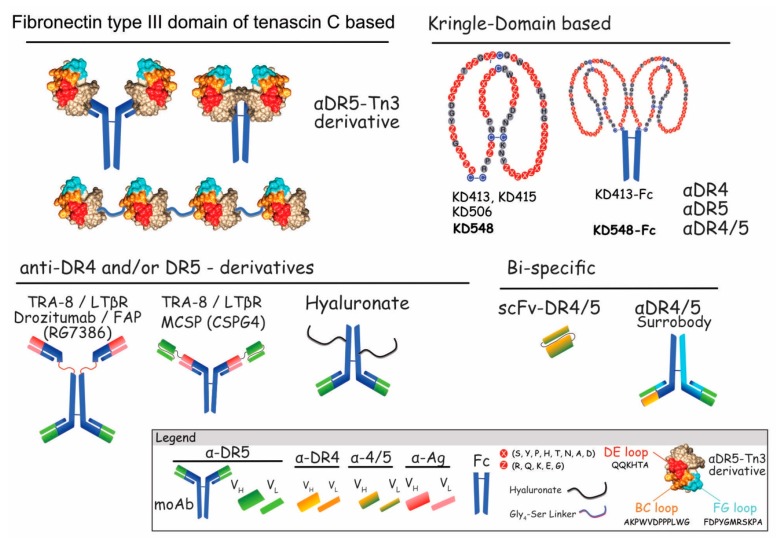
Schematic representation of unconventional and BsAbs harboring selectivity for DR4 and/or DR5. Fibronectin type III domain of tenascin C-based formats recognizing DR5, are presented in the upper left, covalently linked to Igs, Fc or as single-chains. Amino acids highlighted in red/orange and pale blue correspond to TN3 variable loops (DE, FG and BC) which can be mutated without changing its globular structure. These correspond to aa that allow binding to DR5. Amino acids shown in beige, depict TN3 amino acids that need to be preserved to maintain the globular structure of the recombinant protein. Upper right panel illustrates Kringle domain (KD)-based formats fused to the Fc. Specific KDs are numbered 413 to 548. Affinity binding for DR4 and/or DR5 is indicated. Lower left panel depicts anti-DR4 and/or DR5–derivatives targeting TRA-8, LTβR, FAP, MCSP, or hyaluronate. BsAbs targeting both DR4 and DR5 are represented in the lower right.

Unconventional antibody-like and bi-specific derivatives targeting both DR4 and DR5, or DR5 and an antigen specifically expressed by a given tumor cell or tumor microenvironment, have been generated and assessed for their ability to trigger tumor-selective killing. They have mostly been produced based on phage display technology [204,205] or from a protein scaffold library based on the Kringle domain structure [206] (Figure 8).

The first category includes unconventional multivalent anti-DR4 and anti-DR5 antibodies, that were generated either from the fibronectin type III tenascin C domain (Tn3) or the Kringle domain (KD) protein scaffold libraries (Figure 8). Interestingly, while anti-DR5-Tn3 covalently linked to Igs displayed pro-apoptotic activity, these unconventional divalent antibodies were far less efficient than TRAIL. However, in line with previous studies demonstrating superior activity of TRAIL functionalized to nanoparticles [64], multivalent DR5-Tn3 linear chains were found to be 10 to 100-fold more potent than TRAIL in vitro and in vivo [205]. Earlier on, another group has developed DR4–DR5 bispecific antibodies (BsAbs) using yeast surface display, based on the Kringle domain (KD). Fused to dual affinity Ig-Fc Kringle, domain-based moAbs showed high-affinity target binding, and enhanced capacity to induce apoptosis and inhibit tumor growth, in vivo, as compared with the standard monomer counter parts [206,207]. Interestingly enough, receptor complex formation analysis comparison after TRAIL or KD548-Fc stimulation revealed that this DR4/5 dual-specific Kringle domain agonist variant induces the recruitment proteins of additional proteins to the TRAIL DISC complex, including riboflavin kinase, nox1, and Rac1, whose activation induces reactive oxygen species (ROS)-mediated cell death [208]. This DR4/5 dual-specific Kringle domain agonist is likely to enter clinical trials soon, as efforts are being made to produce it at high levels [209]. Although, preliminary, these results highlight the potential therapeutic benefits that can be achieved by targeting both receptors, rather than targeting specifically one of the two death receptors. Several other bi-DR4/5-specific antibody derivatives have been described. Using the phage display technology, MedImmune developed numerous scFv constructs selective for DR4 and/or DR5 [204]. Out of the published screen, 10 anti-DR4 scFv and 6 anti-DR5 scFv were selected. Each of them exhibited specificity towards their own target, but these scFvs displayed only mild anticancer properties in vitro, in the presence of cycloheximide. Strikingly, albeit DR4 and DR5 show high sequence homology, and despite the fact that the main homologous amino acids are accessible as immunogen, as inferred from the crystallographic structure of DR4 and DR5 (Figure 2), very few scFvs—less than 2%—displayed crossreactivity for both receptors. A second screen, alternating rounds of selection on either DR4 or DR5, enabled the identification of 134 distinct dual-DR4/5-scFvs. However, none of these scFvs were able to induce cell death in the two cell lines tested, HT1080 and ST486, a fibrosarcoma and a Burkitt’s lymphoma, respectively [204]. This library, nonetheless, probably represents an interesting source of scFvs for the engineering of diabodies or whole antibodies targeting solely DR4, DR5, or both (Figure 8). Using a similar approach, namely a human phage display surrobody library, an antibody crossreactive to both DR4 and DR5 was found [210]. However, this antibody displayed moderate antitumor potential in vitro, since protein-G beads had to be used prior incubation with the target cells to detect its antitumoral activity. Yet, this surrobody exhibited potent activity in vivo [210], most likely due to ADCC.

Other bispecific derivatives described, so far, are based on tetravalent antibodies (Figure 8). These harbor TRA-8 or Drozitumab variable chains and scFv fragments targeting either LTβR or MCSP, also known as lymphotoxin β receptor and melanoma associated chondroitin sulfate proteoglycan, respectively. BsAb TRA-8xLTβR antibodies are aimed at targeting epithelial cancer cell lines [211]. Constructed with a scFv:LTβR fused to the *N*- or the *C*-terminus of the heavy chain of TRA-8 antibody (Figure 8), both BsAbs inhibited tumor growth of LTβR-expressing cells in vivo. More recently, a MCSPxDR5 bispecific antibody, engineered from a high affinity MCSP moAb to which the variable binding domains of tigatuzumab were covalently linked, was assessed for antitumor selectivity towards melanomas [212]. MCSP is a well-established target for melanoma immunotherapy, since it is overexpressed in more than 90% of melanomas, but its expression is restricted in normal melanocytes. By targeting this membrane-bound protein with the BsAb MCSP × DR5, the authors of this study have been able to demonstrate first that binding of MCSP × DR5 mainly occurs on MCSP + DR5 + cells, compared to MCSP − DR5 + cells, indicating that binding mainly occurs through MCSP, and second, that BsAb MCSP × DR5 is able to induce tumor cell death both in an ADCC-dependent and -independent manner, in vitro [212]. Consequently, MCSP × DR5 represents an interesting compound for melanomas.

The last category of BsAbs has been engineered with scFvs or ligands selective for a given antigen present in the tumor microenvironment and scFv:DR5. It includes RG7368, a fibroblast-activation protein (FAP)-targeted DR5 bispecific antibody derived from Drozitumab [213]. RG7368 was found to target both tumor associated fibroblasts from the stroma, and DR5 present on tumor cells. This strategy permits to put in contact anti-DR5 agents in FAP-positive tumoral microenvironment. RG7368 was found to induce potent tumor killing in a FAP-dependent manner, and to inhibit tumor growth in vivo [213]. Combining RG7368 with irinotecan or doxorubicin also demonstrated a substantial increase in tumor growth regression in colorectal patient-derived xenografts. Another tetravalent scFv:DR5-based hybrid antibody has recently been evaluated, with success, for its ability to treat liver metastasis in preclinical animal xenograft models [214]. Multivalency was afforded in this study by the covalent functionalization of hyaluronate (HA) to scFV:DR5, enabling binding to receptor for hyaluronan-mediated motility (RHAMM) and CD44, two receptors significantly overexpressed in a variety of tumors.

### 5.3. Chimeric Antigen Receptors (CARs)

**Figure 9:**
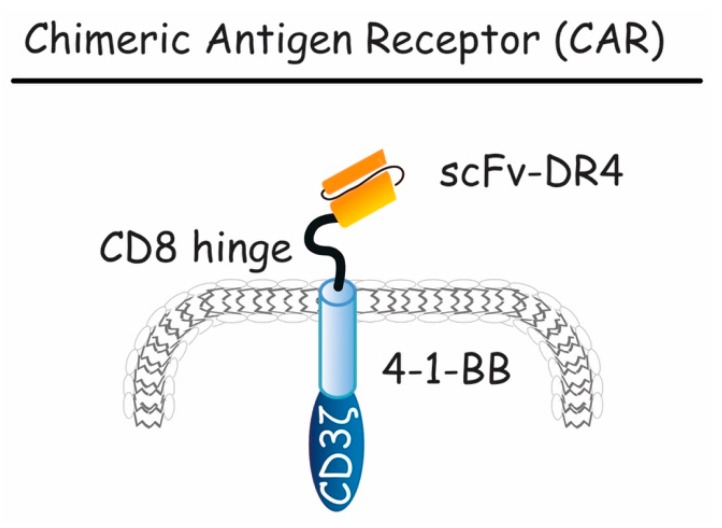
Schematic representation of chimeric antigen receptor (CAR). Here, the CAR construct is presented, containing a scFv extracellular domain targeting DR4, a CD8 hinge and a CD3 intracellular domain mediating T-cell activation. This compound is capable of activating both DR4-mediated apoptosis and tumor specific T-cell cytotoxicity.

Chimeric antigen receptors are promising tumoricidal formats. They are designed by a scFv recognizing specific tumor antigen fused to an intracellular T-cell activation system [215,216]. Upon contact with target antigen, CARs induce T-cell signaling functions, such as cytokine secretion, enhanced cell proliferation and survival, and improves effector cell functions [217]. To date, only one CAR targeting TRAIL death receptors has been described (Figure 9). This TR1-scFv-CAR was developed using scFv recognizing DR4 [218]. Expressed on the cell surface of DR4-deficient Jurkat cells, as well as a NK cell line or human peripheral blood cells (PBLs), it induced DR4-mediated apoptosis of target cells expressing DR4 endogenously. In addition, when expressed in immune cells, NK, and PBLs, respectively, TR1-scFv-CAR induced CAR-mediated cytolytic activity, demonstrating that CARs targeting DRs could be used as anticancer therapeutic tools to induce tumor regression [218]. Along the line, smart CAR systems based on combinatorial antigen recognition [219] are likely to offer ideal tools to deliver, specifically, TRAIL or TRAIL derivatives to the tumor.

### 5.4. Antibody Drug Conjugate-Like TRAIL Derivatives (ADC-Like)

**Figure 10:**
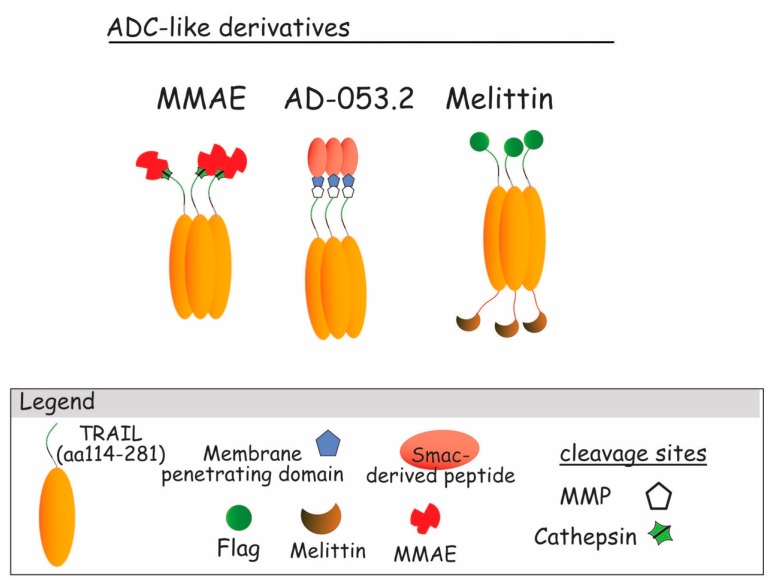
TRAIL ADC-like derivatives. From the left to the right : ADC-like TRAIL build up with Smac-derived peptide (AD-053.2) an intracellular pro-apoptotic agent [225]; Melittin ADC-like, an antibacterial highly cytotoxic harbored on trimeric TRAIL and linked with FLAG [226,227]; MMAE–TRAIL, TRAIL trimeric molecule linked to the monomethyl auristatin E, an antimitotic agent [222,223,224].

The ADC technology takes advantage of innovative chemistry or biochemistry, and of high specificity of antibodies for their target antigens, to deliver a more powerful cytotoxic agent to specific tumor cells [220,221]. Although this method has, so far, never been tested with moAb or derivatives targeting DR4 or DR5, it has been assessed with recombinant TRAIL variants to overcome chemotherapy related toxicity, as well as TRAIL resistance. It has been used for example to conjugate TRAIL with monomethyl auristatin E (TRAIL–MMAE) via valine and citrulline linkage [222,223] (Figure 10) or PEG [224]. Selective targeting of the tumor with TRAIL allowed selective delivery of the cytotoxic drug MMAE to the cancer site. After binding, TRAIL–MMAE is rapidly internalized into the cytoplasm of targeted cancer cells, and released in lysosome via lysosomal-specific cleavage of the linker, placed between TRAIL itself and MMAE. Once released into the cytoplasm, the cytotoxic drug exerts its antimitotic action, leading to cancer cell death. The study of proof-of-concept, demonstrated, in vitro, that the human TRAIL-resistant breast carcinoma cell line MCF-7, efficiently internalized MMAE into its cytoplasm, and died by apoptosis. Interestingly, in vivo, TRAIL–MMAE exhibited a long half-life (>11 h) and displayed potent antitumor activities in xenograft models, while remaining safe for the animal, since no sign of toxicity could be detected [223]. The PEGylated TRAIL–MMAE conjugate was also found to display good pharmacodynamics, with a half-life reaching 7 h. Interestingly, it exhibited selective antitumoral potential, but no signs of hepatotoxicity [224]. AD-O53.2 is another ADC-like TRAIL derivative (Figure 10). It has been engineered by fusing TRAIL and Smac/Diablo with a linker containing a metalloprotease cleavage site and a membrane penetrating peptide, with the aim to overcome cancer cell resistance to TRAIL-induced cell death [225]. AD-053.2 increased, in vitro, tumor cell sensitivity to apoptosis by 3 to 6 orders of magnitude, and was very potent in inducing tumor regression in xenograft animal models [225]. Last, an ADC-like TRAIL derivative based on Melittin, a water-soluble 26-amino acid peptide derived from bee venom of *Apis mellifera*, has been tested for its capacity to induce tumor cell killing. Melittin, for unknown reasons, exerts selective cytotoxicity on a large variety of tumor cells, but is not toxic for normal cells. It had previously been found to sensitize apoptosis induced by TRAIL in hepatocellular carcinomas [226]. Fused to TRAIL in an ADC-like fashion, TRAIL–Melittin conjugate was found, in vitro, to display a moderate increase in antitumor properties, as compared to TRAIL alone [227]. With the exception of the TRAIL–Melittin conjugate, whose gain of function was rather limited, these findings suggest that conjugation of DR4 or DR5 derivatives in an ADC-like antitumoral drug could also lead in the future to efficient TRAIL therapies, provided that such a compound remains safe in vivo, but efficient in inhibiting tumor growth.

## 6. Conclusions

The discovery of TRAIL and its receptors in the mid-’90s raised much interest for antitumor therapies, prompting preclinical and clinical evaluation of TRAIL or TRAIL receptor agonist antibodies. While early clinical evaluation of these first generation of compounds failed to demonstrate enough efficacy to warrant further development, the increase in understanding of the molecular events governing TRAIL-mediated apoptosis signal transduction, together with the advent of innovative biomolecular engineering, suggest that targeting TRAIL receptors is still likely to hold promise for cancer therapy.

Likewise, second generation of TRAIL recombinant proteins fused to Fc, scFv, single chains or monoclonal antibodies targeting TRAIL receptors functionalized to nanoparticles or linkers, in order to increase their valency, are being developed with the aim to increase both bioavailability and efficacy. Whereas, it still remains unknown whether DR4 and DR5 targeting therapeutics will meet the requirements to get approval for cancer therapy, a number of these novel formulations raise hope, due to the excellent results obtained from preclinical or early clinical studies.

The next generation of DR4 and DR5 targeting agents are, thus, likely to put hope back on the trail of antitumoral therapeutics.

## Abbreviations

Aa	amino acids
ADC	antibody-drug conjugate
ADCC	antibody-dependant cell-mediated cytotoxicity
APO	apoptosis antigen
Bcl-2	B-cell lymphoma 2, apoptosis inhibitor
BsAbs	bispecific antibodies
CARs	chimeric antibody receptors
CD	cluster of differentiation
CDC	complement-dependent cellular cytotoxicity
c-FLIP	cellular FLICE (FADD-like IL-1β-converting enzyme)-inhibitory protein
CPT-TRAIL	circularly permuted TRAIL
DC	dendritic cell
DcR	decoy receptor
DcR1	decoy receptor 1, TRAIL-R3
DcR2	decoy receptor 2, TRAIL-R4
DD	death domain
DISC	death-inducing signaling complex
DR	death receptor
DR4	death receptor 4, TRAIL-R1
DR5	death receptor 5, TRAIL-R2
Fab	fragment antigen-binding
Fas	fibroblast associated surface antigen
Fas-L	as ligand
Fc	crystalized fragment
FcR	crystalized fragment receptor
GPI	glycosylphosphatidylinisotol
KD	Kringle domain
Mcl-1	myeloid cell leukemia-1
moAbs	monoclonal antibodies
NK cell	natural killer cell
OPG	steoprotegerin, TRAIL-R5
PBLs	eripheral blood cells
ROS	reactive oxygen species
rhTRAIL	human recombinant TRAIL
scFv	single chain fragment variable
sc-TRAIL	single chain TRAIL
sTRAIL	soluble TRAIL
TNC-TRAIL	tenascin-C-TRAIL
TNF	tumor necrosis factor
TNF-α	tumor necrosis factor alpha
TRAIL	TNF-related apoptosis-inducing ligand
